# Individual- and Regional-level determinants of Human Papillomavirus (HPV) vaccine refusal: the Ontario Grade 8 HPV vaccine cohort study

**DOI:** 10.1186/1471-2458-14-1047

**Published:** 2014-10-08

**Authors:** Olivia Remes, Leah M Smith, Beatriz E Alvarado-Llano, Lindsey Colley, Linda E Lévesque

**Affiliations:** Department of Public Health Sciences, Queen’s University, 21 Arch Street, Room 313, Kingston, Ontario Canada; Department of Epidemiology, Biostatistics, and Occupational Health, McGill University, Montreal, Quebec Canada

**Keywords:** Human papillomavirus, HPV vaccine, Cohort studies, Vaccine coverage, Vaccine hesitancy, Cohort studies

## Abstract

**Background:**

Studies on the determinants of human papillomavirus (HPV) vaccine use have generally focused on individual-level characteristics, despite the potentially important influence of regional-level characteristics. Therefore, we undertook a population-based, retrospective cohort study to identify individual- and regional-level determinants of HPV vaccine refusal (non-receipt) in Ontario’s (Canada) Grade 8 HPV Immunization Program.

**Methods:**

Ontario’s administrative health and immunization databases were used to identify girls eligible for free HPV vaccination in 2007–2011 and to ascertain individual-level characteristics of cohort members (socio-demographics, vaccination history, health care utilization, medical history). The social and material characteristics of the girl’s region (health unit) were derived from the 2006 Canadian Census. Generalized estimating equations (binomial distribution, logit link) were used to estimate the population-average effects of individual- and regional-level characteristics on HPV vaccine refusal.

**Results:**

Our cohort consisted of 144,047 girls, 49.3% of whom refused HPV vaccination. Factors associated with refusal included a previous diagnosis of Down’s syndrome (OR = 1.37, 95% CI 1.16-1.63) or autism (OR = 1.60, 95% CI 1.34-1.90), few physician visits (OR = 1.45, 95% CI 1.35-1.55), and previous refusal of mandatory (OR = 2.23, 95% CI 2.07-2.40) and optional (OR = 3.96, 95% CI 3.87-4.05) vaccines. Refusal was highest among the lowest and highest income levels. Finally, a previous diagnosis of obesity and living in an area of high deprivation were associated with lower refusal (OR = 0.87, 95% CI 0.83-0.92 and OR = 0.82 95%, CI 0.79-0.86, respectively).

**Conclusions:**

Studies on HPV vaccine determinants should consider regional-level factors. Efforts to increase HPV vaccine acceptance should include vulnerable populations (such as girls of low income) and girls with limited contact with the healthcare system.

**Electronic supplementary material:**

The online version of this article (doi:10.1186/1471-2458-14-1047) contains supplementary material, which is available to authorized users.

## Background

The human papillomavirus (HPV) is the most common sexually transmitted infection worldwide, with an estimated 75% of all women having at least one HPV infection during their lifetime [1]. One of the most common manifestation of HPV infections is genital warts, with a prevalence of approximately 1-4% among young, sexually active women [2]. Importantly, the presence of anogenital warts can lead to significant physical, emotional, and social problems for those affected [3]. While genital warts inflict the greatest HPV-related burden on youth, HPV infections may also lead to cancers of the anogenital tract in adulthood, the most frequent being cervical cancer. Of the over 100 types of HPV that have been identified, HPV types 6 and 11 cause 90% of cases of genital warts [4] and types 16 and 18 cause 70% of cases of cervical cancer worldwide [5, 6]. Overall, infections with these four HPV types represent a significant burden of illness and distress, and are estimated to cost the Canadian healthcare system $33 million annually [7].

In 2006, the quadrivalent HPV (qHPV) vaccine was approved for use in Canada, the United States, Europe, and elsewhere [8]. In view of the vaccine’s high efficacy, and the public health implications of HPV-related diseases, the Canadian government allocated $300 million to the provinces and territories to fund the first three years of a national, school-based HPV vaccination program [9]. Despite widespread anticipation about the vaccine’s potential to reduce the burden of anogenital warts and cervical cancer, HPV vaccine coverage was generally lower than anticipated in Canada, reaching nationwide lows of 50% in Alberta and Manitoba, 53% in Ontario, and 65% in British Columbia [10, 11]. High refusal of the HPV vaccine has also been documented in a number of European and Indo-Pacific countries, the United States, and some Central and South American countries [12].

Vaccine refusal falls within the spectrum of a growing phenomenon recently referred to as “vaccine hesitancy” [13]. Decision-making with respect to health behaviours, including vaccination, has been shown to be complex, and influenced by both individual- and regional-level factors [14, 15]. Indeed, the World Health Organization’s (WHO) Strategic Advisory Group of Experts (SAGE) on Immunization recently highlighted the importance of contextual factors including socio-cultural and environmental ones, as determinants of vaccine hesitancy [13]. Yet, studies of the determinants of HPV vaccine uptake published to date have focused predominantly on individual-level characteristics, with little consideration for the potentially important influence that an individual’s environment can have on the decision-making process [16, 17]. Given that low vaccine coverage undermines public health efforts aimed at reducing the burden of HPV-related diseases, and may threaten the cost-effectiveness of HPV vaccination programs, we undertook a population-based, retrospective cohort study of girls eligible for Ontario’s Grade 8 HPV vaccination program to identify the individual- and health unit-level determinants of HPV vaccine refusal.

## Methods

This study was approved by the Research Ethics Board of Queen’s University and Sunnybrook Health Sciences Centre.

### Ontario’s Grade 8 HPV vaccination program

Ontario’s HPV vaccination program was initiated in September of 2007. This voluntary program offers three doses of the vaccine free-of-charge to all Grade 8 girls in the province. These doses are generally administered by Public Health Nurses at school clinics in September/October, November/December, and March/April to correspond with the recommended 0-, 2-, and 6-month dosing schedule of the vaccine. Although the vast majority of doses are given at these school clinics, eligible girls may also receive the vaccine through their public health unit or family physician. At the time of this study, eligible girls had until the end of their Grade 9 year to complete the vaccination series, provided they initiate it in Grade 8 [17]. All doses of the HPV vaccine are documented in the Immunization Record Information System (IRIS) database, regardless of where the dose is administered. Over 80,000 girls are eligible for this program each year.

### Data sources and record linkage

Four administrative health databases were used for this study: (1) the Registered Persons Database (RPDB) to identify the birth cohorts and obtain information on socio-demographics, (2) the Canadian Institute for Health Information’s Discharge Abstract Database (CIHI-DAD) for dates of hospital admissions and discharge diagnoses, (3) the National Ambulatory Care Reporting System (NACRS) for information on emergency department visit dates and diagnoses, and (4) the Ontario Health Insurance Plan (OHIP) database for information on fee-for-service claims submitted by physicians, including service dates and diagnoses. In these databases, each Ontario resident covered by the province’s universal health insurance plan is represented by a unique encrypted identifier that enables complete record linkage at the level of the individual across databases and over time. These databases are continuously updated and can be accessed through the Institute for Clinical Evaluative Sciences’ (ICES) satellite unit located at Queen’s University. Described elsewhere in detail [18–22], these databases have been used extensively in health research, including studies of the HPV vaccine [23, 24].

To obtain vaccination history, we used the IRIS database, which was developed by the Ministry of Health and Long-Term Care (MOHLTC) to assist the province’s 36 health units in tracking and recording immunizations of school-aged children, as required under the *Immunization of School Pupils Act (1982)* [25]. This database is also used to document the receipt of optional vaccines, including the HPV vaccine. Indeed, the IRIS database has been shown to accurately capture information on HPV vaccination status with a sensitivity of 99.8% (95% CI: 99.3 - 99.9) and specificity of 97.7% (95% CI: 96.3 - 98.7) [26]. At the time of this study, the IRIS database of 21 health units had been transferred to ICES and were available for our use.

We also used data from the 2006 Canadian Census, a mandatory, self-reported survey conducted every five years by Statistics Canada to enumerate all residents of Canada and provide a socio-demographic portrait of the Canadian population. We used the Census database to ascertain socio-demographic information at the level of the health unit (i.e., regional-level characteristics) [27].

### Study design and population

We identified a retrospective cohort of girls eligible for Ontario’s Grade 8 HPV vaccination program between 2007/08 and 2010/11 using the province’s administrative health records. Because a girl’s school grade was not available in these data holdings, birth cohorts were used to identify the eligible population. Since students typically turn thirteen by December 31 of their Grade 8 school year, girls born in 1994, 1995, 1996 and 1997, would have been in Grade 8 in September 2007, 2008, 2009, and 2010, respectively, and would have been eligible for the corresponding year’s vaccination program. Although this approach could miss girls who were advanced or held back a grade, a re-abstraction study of a medium-sized health unit demonstrated that this birth cohort definition correctly identified 96.4% of eligible girls [28]. Girls whose vaccination records were not available at the time of the analysis (i.e., IRIS data not yet transferred to ICES for record linkage) were excluded from the cohort. Cohort members were followed from September 1 of their Grade 8 year until their date of death or March 31, 2011 (study end).

### HPV vaccination status

The outcome of interest was HPV vaccine non-receipt (which we refer to as “refusal”), which was obtained from the IRIS database. A girl was classified as a “refuser” if she received no doses of the HPV vaccine; otherwise she was classified as an “acceptor”.

### Individual-level characteristics

We determined the socio-demographics, vaccination history, medical history, and health care utilization history of cohort members using the administrative health and immunization databases previously described. The RPDB was used to obtain information on age, sex, neighbourhood income quintile, and urban/rural residency at cohort entry.

Vaccination history was derived from the IRIS database and included vaccinations received from birth to cohort entry. The optional vaccines under consideration were hepatitis B and meningococcal conjugate because they are both offered in Grade 7 through a publicly funded, school-based program. Mandatory vaccines included the measles, mumps, rubella (MMR) vaccine, as well as the diphtheria, polio, and tetanus (DTP) vaccine. Vaccination history, particularly with optional vaccines, was used as a proxy for parental/guardian beliefs and attitudes toward vaccines in general.

Medical and health care utilization histories of cohort members were obtained between birth and cohort entry using the physician services (OHIP), emergency department (NACRS), and hospital (CIHI-DAD) databases. The medical conditions considered included those resulting in frequent contact with the health care system, as well as those serious enough to potentially affect the decision to vaccinate against HPV (e.g., autoimmune disorders, cancer, congenital anomalies, heart disease, neurological disease; Additional file 1). Health care utilization was determined based on the number of outpatient physician visits, emergency department visits, hospital admissions, and in-patient length of stay prior to cohort entry. The frequency of each type of utilization was categorized based on the frequency distribution of the data. These categories were used as indicators of an individual’s health status and their propensity to use the health care system.

### Characteristics of the health units

The 2006 Canadian Census was used to obtain information on the social and material characteristics of the health unit within which a cohort member resided at cohort entry. We used information from the 2006 Census since this represented the latest data available prior to cohort entry (i.e., preceding the decision to vaccinate). Since health units are responsible for the administration and delivery of the HPV vaccination program in Ontario, the health unit was chosen as the regional level of interest for this study. Based on a review of the literature on the determinants of HPV vaccine acceptance, we initially considered 19 census variables (Additional file 2). We examined the variability of these characteristics across health units, the collinearity between variables, and each variable’s univariable association with the outcome. Based on these analyses, we determined that only six of these characteristic warranted further consideration - average income, level of education, employment/population ratio, marital status, and living alone.

To further address the potential for collinearity between indicators, as well as to reduce the number of variables contributing to the model (given the limited number of health units contributing to the analysis), we developed an *area deprivation index*. The index was modelled after the Pampalon social and material deprivation index [29] and was derived using principal component analysis (PCA) [30]. Although we included the same six social and material factors contained in the Pampalon index in our PCA, only five loaded as a component/dimension and were thus included in our index; single parenthood was not considered further (Additional file 3). A score was calculated for each health unit based on the factor loadings of each of the five indicators. For the purposes of analysis, these scores were subsequently categorized into quartiles.

### Statistical analysis

To estimate the population average effect of the determinants of interest on HPV vaccine refusal, we used generalized estimating equations (GEE) to account for the correlation introduced by the clustering of individuals within health units. The characteristics of the health unit were attributed to the girl, making her the unit of analysis. Since the outcome was dichotomous (refusal *vs.* non-refusal), and the correlation between subjects in health units was assumed to be equal, we used GEE with a logit link and an exchangeable correlation structure to estimate odds ratios (ORs) and 95% confidence intervals (CIs; based on robust standard errors). The final model was selected using backward selection and a significance threshold of 0.1 for variable retention.

## Results

Within the 21 health units, we identified 144,047 girls eligible for Ontario’s Grade 8 HPV vaccination program between 2007/08 and 2010/11. Girls were a mean of 13.2 years of age at cohort entry and were followed for an average of 2.6 years. Approximately 3% of cohort members refused MMR and/or DTP vaccination and 42% refused hepatitis B and/or meningococcal conjugate vaccination (Table 1). Almost 65% of girls resided in areas of low material and social deprivation.

**Table 1:** **Baseline characteristics of cohort members (N = 144,047)**

Characteristic	Frequency (%)
**Individual-level characteristics**	
**Socio-demographics**	
Income quintiles	
*1* ^*st*^ *(Low)*	21,357 (15.0)
*2* ^*nd*^	25,689 (17.8)
*3* ^*rd*^	30,823 (21.4)
*4* ^*th*^	32,495 (22.6)
*5* ^*th*^ *(High)*	30,803 (21.4)
*Missing*	2,880 (2.0)
Place of residence	
*Rural*	21,301 (14.8)
*Urban*	119,866 (83.2)
*Missing*	2,880 (2.0)
***Vaccination history***	
Refusal of mandatory vaccines^*^	4,160 (2.9)
Refusal of optional vaccines^†^	60,447 (42.0)
**Health care utilization**	
Hospitalizations	
*≤1*	110,167 (76.5)
*2-4*	29,350 (20.4)
*≥4*	4,530 (0.03)
Inpatient hospital stay (days)	
*≤2*	103,292 (71.7)
*3-1)*	38,359 (26.6)
*≥11*	2,396 (1.7)
Emergency department visits	
*None*	48,413 (33.6)
*1-4*	69,796 (48.5)
*5-12*	21,229 (14.7)
*≥13*	4,609 (3.2)
Outpatient physician visits	
*≤42*	36,861 (25.6)
*43-116*	74,797 (51.9)
*117-206*	26,794 (18.6)
*≥207*	5,595 (3.9)
**Medical history**	
Congenital anomalies	4,723 (3.3%)
Viral diseases	95,070 (66.0%)
Cardiovascular disease	26,620 (18.5%)
Obesity	6,264 (4.4%)
Autism	599 (0.42%)
Mental illness	44,588 (31.0%)
Neurological diseases	12,972 (9.0%)
Down’s syndrome	639 (0.4%)
Diseases of the musculoskeletal system	48,670 (33.8%)
Respiratory disease	63,250 (43.9%)
Diseases of the endocrine system	5,460 (3.8%)
In-situ carcinoma and benign lesions	13,649 (9.5%)
Cancer	2,491 (1.7%)
Malnutrition	6,558 (4.5%)
Immune-mediated disorders	100,874 (70.0%)
**Health unit-level characteristics**	
**Index of area deprivation (quartiles)**	
*1* ^*st*^ *(low)*	93,093 (64.6)
*2* ^*nd*^	21,249 (14.6)
*3* ^*rd*^	18,482 (12.8)
*4* ^*th*^ *(high)*	11,223 (7.8)

*Includes measles, mumps, rubella (MMR) and diphtheria, tetanus, pertussis (DTP) vaccines.

^†^Includes hepatitis B and meningococcal conjugate vaccines.

The average number of cohort members per health unit was 6859 (range: 1012–18171). Overall, 49.3% (n = 71,048) of cohort members refused HPV vaccination during the study period; refusal varied from 41.8% to 60.3% across health units. While absolute differences were small, neighbourhood income quintile was a statistically significant determinant of HPV vaccine refusal, with the highest level of refusal in the highest and lowest quintiles (Table 2). Immunization history was also a determinant of HPV vaccination in that HPV vaccine refusal was more common among girls who had not received optional and mandatory immunizations in the past (OR = 3.96, 95% CI 3.87-4.05; OR = 2.23, 95% CI 2.07-2.40, respectively). Refusal was generally similar across categories of health care utilization and history of a serious medical condition, but there were some exceptions. In particular, there was a dose–response trend with respect to physician visits, whereby girls with the lowest level of contact with a physician were the most likely to refuse HPV vaccination (OR = 1.45, 95% CI 1.35-1.55) and refusal decreased with increasing levels of physician visits (p < 0.05). In addition, parents/guardians of girls with a history of autism or Down’s syndrome appeared more likely to refuse HPV vaccination than caregivers with unaffected children (OR = 1.60, 95% CI 1.34-1.90; OR = 1.37, 95% CI 1.16-1.63, respectively). In contrast, obesity was associated with decreased refusal (OR = 0.87, 95% CI 0.83-0.92). Finally, health unit-level social and material deprivation was associated with HPV vaccine decision-making, with the lowest refusal observed among girls living in regions with the highest level of deprivation (OR = 0.82, 95% CI 0.79, 0.86).

**Table 2:** **Determinants of HPV vaccine refusal**

Characteristic	Refusal ***n (%)***	Unadjusted OR (95% CI)	Adjusted ^†^ OR (95% CI)
**Individual-level characteristics**			
**Income quintiles** ^*^			
1^st^ (low)	21,357 (51.6)	1.24 (1.20, 1.29)	1.13 (1.09, 1.17)
2^nd^	25,689 (47.3)	1.04 (1.01, 1.08)	1.01 (0.98, 1.05)
3^rd^ (reference)	30,823 (46.2)	1.00	1.00
4^th^	32,495 (48.3)	1.09 (1.05, 1.12)	1.12 (1.08, 1.15)
5^th^ (high)	30,803 (49.7)	1.15 (1.11, 1.18)	1.21 (1.17, 1.25)
**Vaccination history**			
Refusal of mandatory vaccines			
No (reference)	139,887 (48.5)	1.00	1.00
Yes	4,160 (78.8)	3.95 (3.66, 4.26)	2.23 (2.07, 2.40)
Refusal of optional vaccines			
No (reference)	83,600 (34.8)	1.00	1.00
Yes	60,447 (69.4)	4.25 (4.16, 4.35)	3.96 (3.87, 4.05)
**Health care utilization**			
Hospitalizations			
≤1 (reference)	110,167 (48.3)	1.00	1.00
2-4	29,350 (49.5)	0.95 (0.93, 0.98)	0.94 (0.91, 0.97)
≥4	4,530 (52.6)	1.13 (1.07, 1.20)	0.96 (0.90, 1.03)
Emergency department visits			
None (reference)	48,413 (50.7)	1.00	1.00
1-4	69,796 (48.9)	0.93 (0.91, 0.95)	1.03 (1.01, 1.06)
5-12	21,229 (47.9)	0.89 (0.87, 0.92)	1.02 (0.98, 1.06)
≥13	4,609 (47.3)	0.87 (0.82, 0.93)	0.99 (0.93, 1.06)
Outpatient physician visits			
≤42	36,861 (55.3)	1.55 (1.46, 1.64)	1.45 (1.35, 1.55)
43-116	74,797 (47.9)	1.15 (1.09, 1.22)	1.24 (1.17, 1.32)
117-206	26,794 (46.1)	1.07 (1.01, 1.14)	1.12 (1.05, 1.19)
≥207 (reference)	5,595 (44.4)	1.00	1.00
**Medical history**			
Congenital anomalies			
No (reference)	139,324 (49.3)	1.00	1.00
Yes	4,723 (50.2)	1.04 (0.98, 1.10)	1.03 (0.97, 1.10)
Viral diseases			
No (reference)	48,977 (52.4)	1.00	1.00
Yes	95,070 (47.8)	0.83 (0.81, 0.85)	0.96 (0.94, 0.99)
Cardiovascular disease			
No (reference)	117,427 (49.5)	1.00	1.00
Yes	26,620 (48.5)	0.96 (0.93, 0.98)	1.03 (1.00, 1.06)
Obesity			
No (reference)	137,783 (49.5)	1.00	1.00
Yes	6,264 (45.0)	0.83 (0.79, 0.88)	0.87 (0.83, 0.92)
Autism			
No (reference)	143,448 (49.3)	1.00	1.00
Yes	599 (64.3)	1.85 (1.57, 2.19)	1.60 (1.34, 1.90)
Mental illness			
No (reference)	99,459 (49.2)	1.00	1.00
Yes	44,588 (49.5)	1.01 (0.99, 1.04)	1.04 (1.01, 1.07)
Neurological disorder			
No (reference)	131,075 (49.3)	1.00	1.00
Yes	12,972 (49.7)	1.01 (0.98, 1.05)	1.06 (1.02, 1.10)
Down’s syndrome			
No (reference)	143,408 (49.3)	1.00	1.00
Yes	639 (60.6)	1.58 (1.35, 1.85)	1.37 (1.16, 1.63)
**Health unit-level characteristics**			
**Index of area deprivation (quartiles)**			
1^st^ (low) (reference)	93,093 (49.8)	1.00	1.00
2^nd^	21,249 (48.9)	0.97 (0.94, 0.99)	1.02 (0.99, 1.06)
3^rd^	18,482 (49.2)	0.98 (0.95, 1.01)	1.01 (0.97, 1.05)
4^th^ (high)	11,223 (46.4)	0.87 (0.84, 0.91)	0.82 (0.79, 0.86)

OR: odds ratio; 95% CI: 95% confidence interval.

^*^Of the 2,880 girls with a missing postal code (and, consequently, income quintile), 92% were classified as refusers. This high percentage most likely reflects emigration from Ontario rather than actual refusal.

^†^Adjusted for all other factors listed in the table.

## Discussion

Nearly half of all girls eligible for Ontario’s free HPV vaccination program between the 2007/08 and 2010/11 school year did not receive the vaccine. Our study showed that factors related to this high level of refusal included previous diagnoses of Down’s syndrome or autism, as well as low income and high income, and infrequent visits to the doctor. Interestingly, obesity and high area deprivation were determinants of HPV vaccine acceptance. Not surprisingly, a history of vaccine refusal was also associated with refusal of the HPV vaccine.

This study is one of the first to consider medical history as a potential determinant of HPV vaccine use. We found that a history of an obesity-related diagnosis was associated with increased HPV vaccine acceptance. As studies indicate there is an important association between obesity and cervical cancer [31], these results are promising as they suggest that this high risk group is receiving protection against cervical cancer. Nevertheless, given then limited information on this topic, these results need to be confirmed.

This study also found that girls with Down’s syndrome or autism were less likely to receive the HPV vaccine than girls who have not been diagnosed with these intellectual disabilities (IDs). However, we used birth year to estimate the girls’ grade 8 school year, and since individuals with IDs are more likely to start school late or be held back school grades [32], it is possible the association we observed merely reflects the fact that these girls were not yet eligible for publicly funded vaccination. Some individuals with IDs may be homeschooled [33], which would limit their access to the school-based vaccination program. Moreover, there is a common misconception that individuals with IDs are less sexually active than their peers [34, 35], which may lead caregivers to perceive HPV immunization as less necessary for these children. In reality, however, adolescents with IDs report similar ages of sexual onset and rates of sexual activity as their typically developing peers [34, 35] and are at higher risk of sexual abuse [36], suggesting they are at a similar (if not higher) risk of HPV-related illnesses. As a result, this association should be further investigated. Regardless, our study highlights the importance of considering medical history in studies on the HPV vaccine as potential determinants of HPV vaccine use and as potential confounders of the effects of HPV vaccination.

We also found that HPV vaccine acceptance increased with increased levels of outpatient contacts with a physician. These results are consistent with studies from the United States that demonstrate the important, positive influence physicians can have on increasing HPV vaccine acceptance among parents and women [16, 37, 38]. Our study suggests that this relationship holds even in the context of a publicly funded, school-based program, where the vaccine is primarily delivered outside of the physician’s office. Nevertheless, it is important to recognize that increased number of physician visits may also be a proxy for parental health beliefs and behaviours, which likely also influence HPV vaccine receipt.

Our study is also one of the first to consider both individual-level and regional-level characteristics as potential independent predictors of HPV vaccination. Interestingly, we found that girls living in areas of high deprivation were more likely to receive the HPV vaccine than girls living in areas of low deprivation. Our results are not consistent with studies from the United States, which report that indicators of deprivation (e.g., low income, low education) are major barriers to HPV vaccination [16]. The latter findings are not surprising given the high cost of the vaccine series (~$450 CND) and the lack of publicly funded vaccination, but are of particular public health importance as these indicators of deprivation are also major risk factors for cervical cancer [39, 40]. Accordingly, our results suggest the publicly funded, school-based nature of the HPV vaccination programs in Canada is helping to reduce inequities in access to the HPV vaccine that may ultimately help reduce inequities in HPV-related illnesses. Further studies are needed to corroborate these findings.

Importantly, our findings on area deprivation were independent of our one individual-level component of deprivation – family income. Indeed, income was associated with HPV vaccine refusal, whereby girls in the highest and lowest income quintiles were the most likely to refuse HPV vaccination. Our finding of high refusal among higher income families is consistent with a study from British Columbia (Canada) that found that parents of higher socio-economic status (SES) were less likely to accept having their daughters vaccinated, despite being more informed about the benefits of the HPV vaccine [41]. Similarly, studies have consistently reported that low income is associated with high refusal [16]. However, most of these studies were conducted in the United States where important financial and access barriers exist. Since financial and access barriers are greatly reduced in countries, like Canada, that offer the HPV vaccine free of charge, our findings suggest that low income may be a proxy for other factors that influence vaccine acceptance in this population. Nevertheless, given the increased vulnerability of girls from low income families to HPV-related diseases (as described above), our findings demonstrate that this continues to be an important group for targeted cervical cancer risk education even in the context of publicly funded vaccination programs.

Finally, we found that HPV vaccine refusal was highest among girls whose parents/guardians had also refused the MMR, DTP, hepatitis B, and meningococcal conjugate vaccines. These findings are consistent with previous studies on factors influencing HPV vaccine acceptance [16, 23, 41] suggesting that caregiver knowledge, values and beliefs regarding vaccination may be a universal determinant of HPV vaccine acceptance, independent of girls’ medical history, health care utilization, and characteristics of the environment in which she lives.

Our study has a number of strengths, including that it benefits from a large, representative sample and validated HPV vaccination data. Nevertheless, there are also a number of limitations that should be considered. First, the factors available for analysis were restricted to those captured by administrative health databases and the Canadian Census. As a result, residual confounding may have been introduced if important determinants were not included in the analysis. In addition, given that we did not have information on individual-level deprivation, we cannot be certain that area deprivation is a determinant of HPV vaccine refusal independent of individual-level deprivation. However, our analysis did account for one important component of individual-level deprivation (i.e., family income), and area deprivation remained an independent predictor. Another limitation is that a girl’s vaccination status would have been misclassified as “refused” if she moved outside of the 21 participating health units during study follow-up and subsequently received her first dose of the vaccine elsewhere. However, given most girls who initiate their vaccination series do so within the first few months following cohort entry, this proportion is likely to be very low. It is also important to recognize that, given the high prevalence of the outcome (vaccine refusal), the odds ratios over-estimate the risk ratios. Finally, the generalizability of our results to jurisdictions is unknown.

## Conclusion

Our study highlights the importance of including characteristics of both the girl and her environment when carrying out studies on HPV vaccination. In addition, our results indicate that certain medical conditions should be further investigated as potential determinants of HPV vaccine use and as potential confounders of the effects of HPV vaccination. Finally, our results support the positive impact physicians may have on HPV vaccine use, even in the context of a publicly funded system, suggesting physicians could play a particularly valuable role in HPV vaccine educational efforts.

## Electronic supplementary material

Additional file 1: **Diagnostic Codes used to identify medical conditions.** (DOC 35 KB)

Additional file 2: **2006 Canada Census variables considered as potential health unit-level determinants of HPV vaccine refusal.** (DOCX 12 KB)

Additional file 3: **Health unit characteristics comprising the Pampalon index.** (DOCX 14 KB)
