# Supplementary material for: Individual- and Regional-level determinants of Human Papillomavirus (HPV) vaccine refusal: the Ontario Grade 8 HPV vaccine cohort study
Source: BMC Public Health. 2014 Oct 8;14:1047. doi: 10.1186/1471-2458-14-1047 (PMC4210569; doi:10.1186/1471-2458-14-1047)
Supplement: Supplementary file 1 — Additional file 1: Diagnostic Codes used to identify medical conditions. (DOC 35 KB) [file 12889_2014_7174_MOESM1_ESM.doc]

**Additional file 1. Diagnostic Codes used to identify medical conditions**

| Medical Condition | **Type of diagnostic Code** | | |
| --- | --- | --- | --- |
|  | **OHIP Code** | **ICD-9 Code** | **ICD-10 Code** |
| Congenital anomalies | 741-746 | 741-746 | Q00-Q07, Q10-Q18, Q20-Q28 |
| Viral diseases | 070, 033, 052, 053, 054, 057, 075, 078, 079 | 570, 573, 050-059 | B15-B19, B00-B09 |
| Cardiovascular diseases | 390-442, 785 | 390-442, 785 | R00-R01, I00-I02, I05-I09, I10-I15, I20-I25, I26-I28, I30-I52, I60-I69, R00-R01 |
| Obesity | 278 | 278 | E66 |
| Autism | 299 | 299 | F84.0, F84.1 |
| Mental illness | 290 – 299, 300-319 | 290-299, 300-319 | F00-F99 |
| Neurological disorders | 320-359 | 320-359 | G00-G09, G20-G26, G35-G37,  G40, G51.0, G70, G71.0 |
| Down’s syndrome | 758 | 758 | Q90 |
| Diseases of the musculoskeletal system | 737, 739, 781 | 737, 739, 781 | M95-M99, T14.4, M20-M25 |
| Respiratory diseases | 010-011, 486, 769 | 010-018, 480-486, 769 | A15-A19, J12-J18, J80, P22 |
| Disorders of the endocrine system | 243, 244, 245, 250 259 | 243, 244, 245, 259  250 | E01.8, E02, E03, E06, E10-E14, E20-E35 |
| In-situ carcinoma and benign lesions | 210-239 | 210-234 | D00-D36 |
| Cancer | 140-209, 180 | 140-209, 235-239, C53 | C00-C97, D37-D48, C53 |
| Malnutrition | 260-269.9 | 260-269.9 | E40-E46 |
| Immune-mediated disorders | 477, 691, 708 | 477, 691, 708 | J30, L20-L30 |
