# Supplementary material for: Individual- and Regional-level determinants of Human Papillomavirus (HPV) vaccine refusal: the Ontario Grade 8 HPV vaccine cohort study
Source: BMC Public Health. 2014 Oct 8;14:1047. doi: 10.1186/1471-2458-14-1047 (PMC4210569; doi:10.1186/1471-2458-14-1047)
Supplement: Supplementary file 2 — Additional file 2: 2006 Canada Census variables considered as potential health unit-level determinants of HPV vaccine refusal. (DOCX 12 KB) [file 12889_2014_7174_MOESM2_ESM.docx]

**Additional file 2. 2006 Canada Census variables considered as potential health unit-level determinants of HPV vaccine refusal**

| **Variable** | **Variable type** | **Included in determinants analysis** |
| --- | --- | --- |
| Lone parent families | Categorical | Yes |
| Average income of people ≥15 year | Continuous | Yes |
| North American Aboriginal ^†^ | Categorical | Yes |
| Registered Indian Status ^†^ | Categorical | Yes |
| Non-family persons living alone | Categorical | Yes |
| Employment – by labour force activity | Categorical | Yes |
| Visible minority status | Categorical | Yes |
| Education level – no certificate, diploma, or degree | Categorical | Yes |
| Marital status – divorced, separated, widowed | Categorical | Yes |
| Rented dwellings | Categorical | No |
| Arabic ethnicity | Categorical | No |
| West Asian ethnicity | Categorical | No |
| South Asian ethnicity | Categorical | No |
| East and South-East Asian ethnicity | Categorical | No |
| Females 10 to 14 years | Categorical | No |
| Non-English mother tongue | Categorical | No |
| Non-French mother tongue | Categorical | No |
| Arabic mother tongue | Categorical | No |
| Chinese mother tongue | Categorical | No |

^†^ Combined to create one variable capturing the percentage of residents in a health unit identifying as being of Aboriginal descent
