# Supplementary material for: Individual- and Regional-level determinants of Human Papillomavirus (HPV) vaccine refusal: the Ontario Grade 8 HPV vaccine cohort study
Source: BMC Public Health. 2014 Oct 8;14:1047. doi: 10.1186/1471-2458-14-1047 (PMC4210569; doi:10.1186/1471-2458-14-1047)
Supplement: Supplementary file 3 — Additional file 3: Health unit characteristics comprising the Pampalon index. (DOCX 14 KB) [file 12889_2014_7174_MOESM3_ESM.docx]

**Additional file 3. Health unit characteristics comprising the Pampalon index**

| *Health unit-level characteristic* | *Statistics Canada Census definition* | *Type of Factor* | *In the original Pampalon index?* | *In the area deprivation index?* |
| --- | --- | --- | --- | --- |
| (Low) education level | Proportion of people aged 15 years and older with no high school diploma, certificate or degree | Material | Yes | Yes |
| Employment /population ratio | Ratio of individuals 15 years of age and older who are employed to the total population 15 years of age and older | Material | Yes | Yes |
| Average income | Average personal (before tax) income of individuals 15 years of age and older | Material | Yes | Yes |
| Living alone | Proportion of individuals 15 years of age and older living alone | Social | Yes | Yes |
| Separated /divorced /widowed | Proportion of individuals 15 years of age and older who are separated, divorced or widowed | Social | Yes | Yes |
| Single parents | Proportion of lone-parent families | Social | Yes | No |
